# Editorial: Surface Electromyography: Barriers Limiting Widespread Use of sEMG in Clinical Assessment and Neurorehabilitation

**DOI:** 10.3389/fneur.2021.642257

**Published:** 2021-02-12

**Authors:** Roberto Merletti, Isabella Campanini, William Z. Rymer, Catherine Disselhorst-Klug

**Affiliations:** ^1^Laboratory for Engineering of the Neuromuscular System (LISiN), Department of Electronic Engineering and Telecommunications, Politecnico di Torino, Turin, Italy; ^2^LAM - Motion Analysis Laboratory, Correggio, Neuromotor and Rehabilitation Department, Azienda Unita' Sanitaria Locale, Istituto di Ricerca e Cura a Carattere Scientifico di Reggio Emilia, Reggio Emilia, Italy; ^3^Shirley Ryan Ability Lab, Single Motor Unit Laboratory, Chicago, IL, United States; ^4^Department of Rehabilitation and Prevention Engineering, Institute of Applied Medical Engineering, Rheinisch-Westfälische Technische Hochschule Aachen University, Aachen, Germany

**Keywords:** surface electromyography, sEMG, HDsEMG, clinical use, cultural barriers, educational barriers, technical barriers, administrative barriers

## Introduction

Bioelectric signals provide an extraordinary opportunity to discover and quantify a wealth of phenomena associated with the organs that generate them. In addition, they provide extensive potentially valuable information for medical doctors (MDs), physiotherapists (PTs), occupational therapists (OTs), and movement scientists (MSs), for functional diagnosis, patient path management, evaluation of patient recovery and progress, and to document or quantify the effectiveness of treatments/trainings.

Clinical researchers and engineers have expected that such information would be highly welcomed by clinicians because of the evidence that such information would add to their rehabilitative activity. This has not been the case for surface EMG (sEMG), a fact that motivated this Frontiers project, as well as the publication of a number of tutorials and consensus papers (1–5), and some EU efforts since 1999 (6). A recent Frontiers e-book illustrated sEMG scientific/technical innovations but did not address the “barrier” problem (7).

This Frontiers Project presents 18 contributions from 7 countries and 80 authors (33 engineers, 16 MDs, 18 PTs and OTs, and 13 MSs) who are highly respected experts in the many fields of sEMG. The general problem is addressed in Campanini, Disselhorst-Klug et al., while results of interviews are presented in Manca et al., Feldner et al., and Cappellini et al. as well as the situation in specific countries (Manzur-Valdivia and Joel Alvarez-Ruf; Portero et al.), and teaching/communication experiences (De la Fuente et al.; McManus et al.). Specific clinical applications concerning neurorehabilitation, gait analysis, sport, kinesiology, exoskeletons, occupational medicine, and ergonomics, are discussed in Cappellini et al., Agostini et al., Felici and Del Vecchio, Goffredo et al., Medved et al., Steele et al., Pilkar et al., Campanini, Cosma et al., Ranavolo et al., and Disselhorst-Klug and Williams.

The disciplines listed are not the top branches of medicine in terms of funding and general attention. Their impact is measured in terms of function recovered, improved quality of life or prevented deterioration, and social costs (Ranavolo et al.; Martin and Acosta-Sojo). These “quantities” are not easy to measure. The focus on the clinical impact of quantitative assessments of movement and of the so called “hard sciences” to the rehabilitation field is relatively recent (8–10).

There is general agreement that the barriers limiting the clinical use of sEMG are cultural/educational and technical/administrative.

## Cultural and Educational Barriers

The education of PTs and OTs varies considerably across countries. France offers a 1+4 year non-academic degree; some countries offer a clinical doctorate (DPT, DOT). Proper interpretation of sEMG requires both technical and clinical competencies. Should these two different types of expertise be merged into a single professional expert, or in two distinct professional figures?

Some universities (TU Delft, Erasmus MC, and LUMC^1^) started training a “clinical technologist” who is a registered healthcare professional carrying out tests and signal processing independently. This new figure has been well-received by the local clinical community (see text footnote 1). Manca et al. indicate that, in reply to their questionnaire, “… the professional figure of ‘human motion analyst’ was put forward by some respondents as a possible reference to manage sEMG assessments in the clinical setting.” Some interviewees in the work of Cappellini et al. made similar propositions.

The alternative option of integrating technical knowledge into the training of PTs, OTs, MSs, and other health operators, finds approval but few implementation efforts. A research group integrating clinicians from Brazil and Chile promoted a Winter School to integrate knowledge about sEMG into the background of clinical operators (De la Fuente et al.). Other authors propose that practical experience working with sEMG should be embedded into education in the form of workshops or course placements to effectively promote its use, and outline a basic tutorial which could be used as a tool for teaching or self-guided learning (McManus et al.). A new medical degree addressing technological issues^2^ has been activated in Italy. Masters in Advanced Technologies in Rehabilitation are currently offered by other institutions to MDs, PTs, OTs, and MSs^3^^,^^4^.

The need for increased technical training of clinical operators and of their educators is generally recognized by all contributors to the project. The publication of open access teaching material^5^, as well as tutorials and consensus papers is in this direction [(1, 2, 4, 5); Felici and Del Vecchio]. However, freely available tutorials are still not sufficient, since “publishing our work in journals is essential—but publication of research is not, by itself, sufficient if our goal is to support clinical practice. People follow the lead of other people they know and trust when they decide whether to take up an innovation and change the way they practice!” (11). Lack of knowledge of books and journals in the field is common.

The lack of a Ph.D. degree in Physiotherapy, preliminary to the academic training of professors, the lack of research and publication record by PTs (12), and the poor technical and instrumental education of operators lead to a vicious cycle that is hard to break:

*No teaching of sEMG applications*→*No clinical competence in the use of sEMG*→*Few clinical publications and no grant requests in the field for large clinical studies on sEMG*→*No experience acquired at the academic level*→*No academic training of qualified professors*→*No teaching of sEMG applications. Conclusion: undemonstrated utility of the method*.

Is the user and caregiver perspective a key untapped resource in the design, implementation, and use of sEMG devices as indicated by Feldner et al.? Probably not for clinical measurements but the perspective of clinical operators should be considered (13).

There is general agreement by clinicians about the potential clinical usefulness of sEMG as shown in Table 2 in [Manca et al.; Cappellini et al.; (13)]. The interviews carried out by these authors indicate that sEMG “provides unique information on neuromuscular function that is not offered by other assessment techniques/tools.” Large consensus was reached in Manca et al., among 80% to 97% of the 35 respondents interviewed as well as among others interviewed by Cappellini et al. who found that “sEMG use was considered to substantially enhance the quality of patient's assessment.” The slow dissemination of this knowledge seems to be a barrier to clinical translation.

Some contradictions are linked to the fact that (as opposed to ECG) visual analysis and interpretation of sEMG is not easy. However, improper muscle coordination and timing (e.g., in gait analysis) can be readily detected visually. Nonetheless, the interpretation of this information requires a thorough comprehension of biomechanics, of the existing boundaries to movement consequent to pathology and, more generally, several years of expertise (Manca et al.; Campanini, Cosma et al.). Lack of time seems to be one of the main reasons behind this barrier (see the section below).

Difficult interpretation of sEMG without specific education/training was reported by 21 out of 28 interviewees (Cappellini et al.) as well as insufficient education/practice during refresher courses (reported by 20/28), and inadequate education and training of PTs and MDs on sEMG (reported by 17/28). This is another vicious cycle since adequate education, in turn, reduces time for learning and clinical application.

## Technical and Administrative Barriers

Most clinical research studies involving sEMG have been carried out by engineers and life-scientists in research centers, on small groups of 10–50 subjects. Larger clinical trials are few because they must be carried out within health institutions where engineers are rarely present and clinical operators may lack the competence, the support, and the time to carry them out (Campanini, Disselhorst-Klug et al.).

This is another barrier leading to the undemonstrated utility of the method. The absence of academic positions implies that research in physiotherapy must be carried out in parallel with the very time demanding routine of traditional clinical procedures. The lack of a clinical market is the cited reason for manufacturing equipment being mostly oriented to the much smaller research market. As a consequence, clinical operators consider sEMG technology limited to research, cumbersome to use, time-consuming, and expensive.

Some users suggest that clinical sEMG systems should be inexpensive and simple to use, and incorporate some intelligence for correcting errors of the user by automatically eliminating power line interference, movement artifacts, or other problems. This raises the following interesting question: should technology adapt, with some internal intelligence and higher cost, to the lack of competence of the users, or should user competence be increased so that technology can be simpler, cheaper, better managed, and controlled?

Unquestionably, there is a need for standard protocols based on extensive clinical trials (as was done for ECG and EEG), but the initial effort of the SENIAM project (6) and its extensions (4, 5, 14) did not trigger subsequent clinical initiatives. Preparing guidelines and protocols requires funding, time, and active participation of MDs, PTs, OTs, and MSs.

On another related issue, the number of publications about methodologies for sEMG analysis is overwhelming and confusing. Twenty-four years ago Hodges and Bui (15) tested 27 methods for determining muscle activity onset time. There are many more today. The number of methods to monitor sEMG spectral changes is also huge, ranging from Fourier to wavelet, and from entropy to fractal analysis. Most of these approaches will have limited meaning to clinicians. There is a need to define a limited number of clinically tested reliable algorithms and best practices to use with (or propose to) trained clinical investigators. In addition, the temptation to address complex problems, such as dynamic sEMG, have produced at least as many approaches, generating additional confusion. Perhaps the teacher's attention should first be focused on well-tested methods for studying relatively simple situations, such as the timing of muscle activation during gait in well-controlled conditions (Disselhorst-Klug and Williams) along with the contribution to clinical decision-making (Campanini, Cosma et al.). Scientific societies should address this issue.

Lack of time seems to be a major multifaceted barrier (Feldner et al.; Martin and Acosta-Sojo). The application and connection of electrodes is indicated as time-consuming. This is no longer true with the use of wireless systems but knowledge about proper electrode positioning is required to promote time saving (14, 16–18). Time is needed for PTs, OTs, and MSs to learn and practice these techniques. Formal academic teaching would reduce time spent in the clinical environment, but the lack of leaders/clinicians devoted to full-time teaching and research (doctoral students, researchers, and associate/full professors) is another main barrier.

Administrative issues are also relevant because all clinical activities are coded and reimbursed or documented by the clinician according to such codes. Only gait analysis is coded in a few countries. Most other sEMG-based investigations are not.

Only a few insurance companies reimburse basic sEMG examinations. Institutional stakeholders should outline the fact that muscle assessment for proper treatment selection would likely generate savings, rather than cost increases (Campanini, Cosma et al.).

As indicated by Martin and Acosta-Sojo “…EMG does not provide information about life-threatening conditions, although it can provide useful information about health- or profit-threatening conditions.”

Furthermore, in stroke patients “surface EMG would supply information for better assessment of deficits as well as rehabilitation progress and/or efficacy” [Martin and Acosta-Sojo; (19)]. In the (not so) long run, personalized treatment based on personalized assessment (Campanini, Cosma et al.) would reduce the weight of ineffective therapies, increase insurance profits, and reduce costs for national health systems.

## Conclusions

Several negative factors, inconsistencies, and contradictions are outlined in the project. On the one hand, rehabilitation clinicians; (a) recognize the value and need for formal education to enable more rigorous and clear evidence highlighting the benefits of sEMG; and (b) assert that the opportunities to pursue it are inadequate due to administrative and time resource limitations. On the other hand, many textbooks (14) and the abundant free resources available [(1, 2, 4–6); see text footnote 5^6^] are not exploited, either in schools or for continuing education. Increasing their quantity and quality has not been as useful as expected. Vicious cycles can be broken; (a) by extending the number of years required for a clinical degree; and (b) by opening opportunities for higher education and research promoted by scientific associations, the EU, and other national/international bodies.

Removing administrative obstacles is equally important to lighten the workload of clinical operators, leaving time for applying and investigating more recent and well-documented techniques. Doing so would promote experience-based technical improvements, prime virtuous cycles incrementing knowledge, applications, market, and reducing the commercial prices of devices.

Introducing new professional figures such as rehabilitation engineers or clinical technologists (see text footnote 1), alongside clinical operators, would substantially reduce (but would not eliminate) the need for technical training of the latter. As rehabilitation technology is rapidly developing, such figures will become necessary very shortly and early training of these operators is certainly appropriate. The clinical responsibilities of these two professional figures should be defined soon. Finally, a common language for proper communication must be available on both sides to understand the information carried by sEMG.

## Author Contributions

All authors contributed equally to the preparation of the Editorial.

## Conflict of Interest

The authors declare that the research was conducted in the absence of any commercial or financial relationships that could be construed as a potential conflict of interest.

## Footnotes

^1^https://www.tudelft.nl/en/2020/3me/june/clinical-technologists-officially-registered-healthcare-professionals/

^2^https://www.healthcarestudies.com/MedTec-School/Italy/Humanitas-University/ and https://www.humanitas.it/news/24675-medtec-humanitas-university-politecnico-milano-formano-medici-del-futuro

^3^https://www.hunimed.eu/course/master-in-advanced-technologies-in-rehabilitation

^4^https://www.rehabtech.polimi.it

^5^www.robertomerletti.it

^6^www.seniam.org
